# Palladium nanoparticle based smart hydrogels for NIR light-triggered photothermal/photodynamic therapy and drug release with wound healing capability†

**DOI:** 10.1039/d2na00897a

**Published:** 2023-02-14

**Authors:** Xiuzhao Yin, Taojian Fan, Nannan Zheng, Jing Yang, Li Yan, Shuqing He, Fujin Ai, Junqing Hu

**Affiliations:** a College of Applied Technology, Shenzhen University Shenzhen 518060 P. R. China; b College of Health Science and Environmental Engineering, Shenzhen Technology University Shenzhen 518118 P. R. China aifujin@sztu.edu.cn; c Shenzhen Bay Laboratory Shenzhen 518132 P. R. China

## Abstract

Tumor recurrence and wound repair are two major challenges following cancer surgical resection that can be addressed through precision nanomedicine. Herein, palladium nanoparticles (Pd NPs) with photothermal and photodynamic therapy (PTT/PDT) capacity were successfully synthesized. The Pd NPs were loaded with chemotherapeutic doxorubicin (DOX) to form hydrogels (Pd/DOX@hydrogel) as a smart anti-tumor platform. The hydrogels were composed of clinically approved agarose and chitosan, with excellent biocompatibility and wound healing ability. Pd/DOX@hydrogel can be used for both PTT and PDT with a synergistic effect to kill tumor cells. Additionally, the photothermal effect of Pd/DOX@hydrogel allowed the photo-triggered drug release of DOX. Therefore, Pd/DOX@hydrogel can be used for near-infrared (NIR)-triggered PTT and PDT as well as for photo-induced chemotherapy, efficiently inhibiting tumor growth. Furthermore, Pd/DOX@hydrogel can be used as a temporary biomimetic skin to block the invasion of foreign harmful substances, promote angiogenesis, and accelerate wound repair and new skin formation. Thus, the as-prepared smart Pd/DOX@hydrogel is expected to provide a feasible therapeutic solution following tumor resection.

## Abstract 2

A postoperative therapeutic nanoplatform of Pd/DOX@hydrogel can efficiently perform near-infrared light triggered photothermal/photodynamic therapy and chemotherapy to kill residual tumor cells, with the potential to accelerate wound healing.

## Graphical abstract

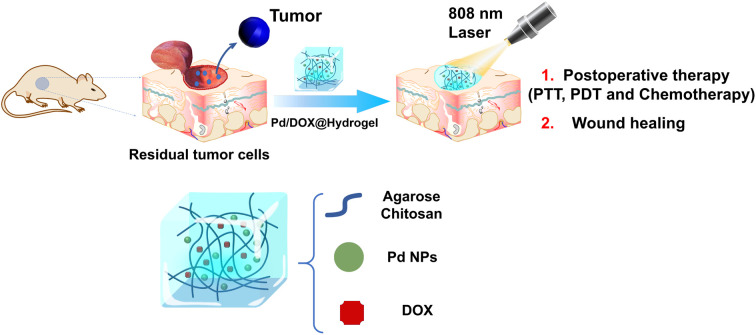

## 1. Introduction

Melanoma is a highly invasive skin cancer originating from melanocytes, which is difficult to cure leading to a major cause of cancer-related death worldwide.^1^ Surgical resection of tumor tissue is still the foremost choice in the clinic among several melanoma treatments.^2^ However, surgery can rarely remove all tumor tissue, and therefore subsequent postoperative treatment, including of residual cancer cells and wound healing, is also a crucial challenge after surgical resection.^3^ Therefore, surgical treatment combined with other treatments need to be explored to further improve therapeutic effectiveness with decreased side effects.

Nanomedicine with multifunctionality makes it possible to develop novel cancer treatment of surgery assisted combined therapy.^4,5^ Among these methods, photothermal therapy (PTT) can treat cancer with good tumor selectivity and avoid the photo-induced toxicity to normal tissue, and are thus promising choices for postoperative therapy. Furthermore, photodynamic therapy (PDT) generates toxic reactive oxygen species (ROS) under light irradiation, which can damage and defunctionalize proteins as well as DNA, resulting in cancer cell death.^6^ However, PDT and PTT are often encountered with limited therapeutic effects due to the hypoxia tumor microenvironment, scattering of light and low tissue penetration depth.^7,8^ In recent years, a combined photothermal and photodynamic effect in a single nanoplatform could demonstrate synergistic anticancer efficacy compared with any single model of PTT or PDT. Some agents even can perform PTT and PDT simultaneously with a single laser,^9,10^ which could receive tremendous attention due to the excellent efficacy. Therefore, it is fascinating to synthesize novel phototherapeutic agents with both high NIR photothermal conversion efficiency and an outstanding photodynamic effect, aiming to achieve superior effectiveness through a combined therapy of PTT and PDT. Among several phototherapeutic agents, Pd NPs as a noble metal material have demonstrated excellent photothermal and photodynamic capability simultaneously, which can perform combined PTT and PDT with photoacoustic (PA) imaging ability.^11,12^ However, Pd NP-based wound dressings for intrinsic PDT and wound healing utilization have rarely been explored, since the direct contact of NPs with wounds can easily cause an inflammatory response of concern in skin tissue.^13^ Besides, nanoparticle-based agents generally have fast biological metabolism and would encounter obvious particle aggregation, and thus they are seldom explored as wound dressings.^14^ Therefore, combinatorial therapy with tissue repair ability as a multifunctional wound dressing for cutaneous melanoma treatment and defective skin tissue repair is extremely needed. Wound dressings can not only control the hemorrhage and promote wound healing by the removal of excess exudates, but also prevent pathogen infection.^15^ To date, wound dressings mainly include gauze, bandages, hydrogels, foam, and smart wound dressings (with stimulus responsive wound dressing and self-healing wound dressing capacity).^16,17^

Polymer-based hydrogels are widely utilized as a promising postoperative wound dressing,^18^ which can absorb tissue exudates and cool the wound surface for relieving the pain of patients.^19^ In addition, hydrogels as an artificial skin repair material were also harnessed to repair cartilage tissue, promoting the regeneration of damaged myocardial tissue.^20^ Additionally, hydrogels with doxorubicin (DOX) loading can achieve acidic microenvironment responsive on-demand controlled drug release.^21^ Thus, hydrogels can be explored as a platform to construct multifunctionality for phototherapy and a drug carrier for cancer postoperative therapy, especially for some skin cancer due to the photon reachability. For effective treatment of melanoma and skin lesions, hydrogel loading with a phototherapeutic agent with skin repair function can be employed to treat tumors and cure skin defects, providing theoretical and technical support for the treatment of clinical melanoma.

**Scheme 1:**
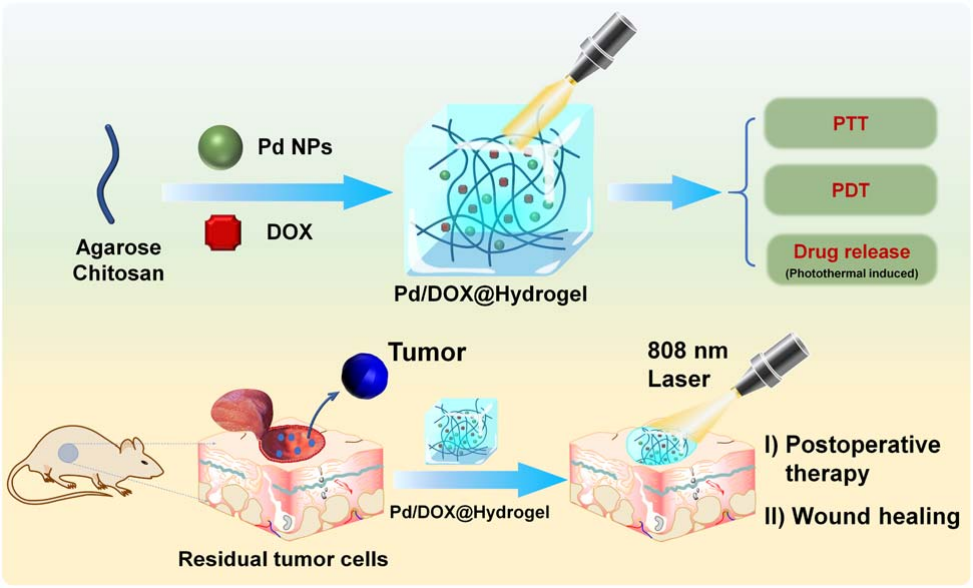
Schematic construction of Pd/DOX@hydrogel for postoperative therapy through NIR-light triggered photothermal/photodynamic therapy and drug release with wound healing capability.

Herein, we developed a chemotherapy drug-loaded hydrogel to explore its potential as a postoperative therapeutic nanoplatform. As shown in Scheme 1, the hydrogel matrix is composed of FDA-approved natural polymers of agarose and chitosan, which can be used as both wound antiseptic dressings and drug-loading reservoirs to achieve sustained drug release. Pd NPs well dispersed in the hydrogel can efficiently convert near-infrared (NIR) light into thermal energy for PTT and to kill residual tumors cells. The hydrogel also has the potential to accelerate wound healing function. Therefore, this study provides an adjuvant therapy system for solid tumor surgery based on Pd NPs: (1) under NIR light irradiation (808 nm), Pd/DOX@hydrogel generates enough heat for PTT, modulating drug release and generating ROS in response to NIR for the further killing of residual cancer cells and (2) Pd/DOX@hydrogel achieved postoperative *in situ* treatment, inhibiting tumor recurrence and wound infection.

## 2. Experimental methods

### 2.1 Materials

Palladium chloride (PdCl_2_, 99%), *N*,*N*-dimethylformamide (DMF, AR, 99.5%), polyvinylpyrrolidone (PVP, MW = 24 000), doxorubicin hydrochloride (DOX), chitosan, and low-melting-point agarose were bought from Sigma Aldrich Chemie GmbH. All chemicals were of at least analytical reagent grade and used without further purification.

### 2.2 Synthesis of Pd nanoparticle

0.02 g PVP was dissolved in 30 mL of DMF under stirring. When the solid powder was completely dissolved, then 0.02 g PdCl_2_ was added slowly. After stirring for 20 minutes, the colorless solution changed to light yellow, and the resulting solution was transferred into a Teflon-lined stainless-steel autoclave, which was heated at 160 °C for 12 h and then naturally cooled to room temperature. The resulting product was washed with deionized water and ethanol alternately several times, and collected by centrifuging at 8000 rpm for 5 min.

### 2.3 Preparation of Pd/DOX@hydrogel

1 mL of glacial acetic acid was dissolved in 100 mL of water to prepare 1% acetic acid solution with stirring. Subsequently, deacetylated chitosan (0.75 g) was added to the above solution with stirring for 6 hours, and 1.5% (w/v) chitosan solution was obtained. Next, 0.2 g of low melting point agarose was added to 10 mL of 1.5% (w/v) chitosan solution at 50 °C for 2 h with stirring, and the resulting hydrogel (2% agarose) was prepared. Pd@hydrogel (containing 200 mg mL^−1^ Pd) was prepared by mixing Pd NP powder with the hydrogel at 50 °C. Pd/DOX@hydrogel was prepared by adding 200 mg mL^−1^ DOX with Pd@hydrogel at 50 °C.

### 2.4 Characterization

The morphologies of the as-prepared samples were analyzed by TEM (JEM-2100F). XRD analyses were performed by using a D/max-2550 PC X-ray diffractometer (Rigaku). The XPS patterns were obtained by using an X-ray photoelectron spectrometer (ESCALab 250). UV-Vis-NIR spectra were recorded by using a UV-1902PC spectrophotometer (Phoenix).

### 2.5 *In vitro* photothermal effect test

In order to evaluate the photothermal effects of Pd NPs, 100 μL of water dispersions with different concentrations of Pd NPs were irradiated with an 808 nm laser at a power density of 0.5 W cm^−2^ for 5 min. Temperature measurement and infrared imaging were performed by using an infrared induction camera.

### 2.6 The detection of singlet oxygen

The extracellular singlet oxygen generation test was carried out by using a 1,3-diphenylisobenzofuran (DPBF) probe. Firstly, the DPBF probe was prepared by mixing 50 mg DPBF dissolved in 50 mL absolute alcohol, and then the DPBF probe was transferred into a light-proof container. Secondly, 2 mL of ethanol was transferred into a colorimetric dish and then added dropwise into 0.4 mg Pd NPs with continuous ultrasonication. Finally, 40 μL DPBF probe was added with stirring for 10 min, and then the solution was irradiated with an 808 nm laser at a power density of 0.5 W cm^−2^ for 1 min, and the absorbance of solution was measured by UV-Vis-NIR spectroscopy. The degree of singlet oxygen production was reflected by the decrease in the absorbance at 410 nm. In the different temperature experiment of DPBF absorption, the above solution was put in a thermostatic water bath for 5 minutes, and then the absorption was measured by UV-Vis-NIR spectroscopy. Temperature was set as: (23 °C, 33 °C, 43 °C, 53 °C, 63 °C, and 73 °C).

### 2.7 Photothermal-induced drug release of Pd/DOX@hydrogel

0.2 mL of Pd/DOX@hydrogel (Pd = 200 μg mL^−1^ and DOX = 200 μg mL^−1^) was added dropwise into a cuvette. Then 1.8 mL PBS buffer was slowly added into it. Pd/DOX@hydrogel at the bottom was irradiated with an 808 nm laser at a power density of 0.5 W cm^−2^ for 5 min, 10 min and 20 min, and the supernatant was measured by UV-Vis-NIR spectroscopy.

### 2.8 Effect of pH on drug release

0.2 mL of Pd/DOX@hydrogel (Pd = 200 μg mL^−1^ and DOX = 200 μg mL^−1^) was added dropwise into a cuvette. Then 1.8 mL PBS buffer was slowly added into it with different pH (pH = 7.4, 6.5 and 5), and the supernatant was measured by UV-Vis-NIR spectroscopy.

### 2.9 Antitumor testing of Pd NPs *in vitro*

Mouse melanoma B_16_F_10_ cells were cultured in DMEM medium supplemented with 10% FBS and 1% PS and then was seeded in 96-well plates (5 × 10^4^ cells per well). The cells were treated with 5 different concentrations (12.5, 25, 50, 100 and 200 μg mL^−1^) of Pd NPs for 24 h. The relative cell viabilities were determined by standard Cell Counting Kit-8 (CCK-8) assay. Similar experimental steps for hydrogel, Pd@hydrogel and Pd/DOX@hydrogel were performed. In addition, B_16_F_10_ cells were seeded in 96-well plates in the same way and were treated with PBS, hydrogel, Pd@hydrogel (200 μg mL^−1^) and Pd/DOX@hydrogel (200 μg mL^−1^ and DOX = 200 μg mL^−1^), respectively. After incubation for 24 h, the medium was removed with calcein-AM/PI for 20 min, and the samples were observed by using a confocal microscope.

### 2.10 *In vitro* biocompatibility assessment of the hydrogel

HDF cells were cultured in DMEM supplemented with 10% FBS and 1% PS and seeded in 96-well plates (5 × 10^4^ cells per well). PBS, hydrogel, Pd@hydrogel (200 μg mL^−1^) and Pd/DOX@hydrogel (200 μg mL^−1^ and DOX = 200 μg mL^−1^) were seeded on HDF cells and incubated for 1, 3, and 5 days in a dark system (5% CO_2_ and 37 °C). At intervals of 1, 3, and 5 days, the cell viability was measured using CCK-8 assay.

### 2.11 Construction of tumor models *in vivo*

Balb/c-nude mice (6–8 weeks old, male) were obtained from Shenzhen Rongwan Biotechnology Co., Ltd. The B_16_F_10_ tumor models were constructed by subcutaneous injection of 5 × 10^5^ cells in 100 μL of DMEM into the flank region of the right front of mice. A 10 mm diameter skin wound was created on the upper part of the tumor when the diameter of the melanoma reached about 4 mm. The melanoma was exposed, simulating a wound caused by surgical resection.

### 2.12 Antitumor therapy strategies

Mice were randomly divided into 7 groups (control, hydrogel, Pd@hydrogel, hydrogel + laser, Pd/DOX@hydrogel, Pd@hydrogel + laser, and Pd/DOX@hydrogel + laser). 50 μL hydrogel was carefully smeared into the solid tumor from day 0 to day 2, while the control group didn't undergo any treatment. Hydrogel + laser, Pd@hydrogel + laser and Pd/DOX@hydrogel + laser groups were irradiated with an 808 nm laser at a power density of 0.5 W cm^−2^ for 15 min. A caliper was utilized to measure the length (*d*) and width (*D*) of the tumor on days 0, 2, 4, 6, 8, 10, 12, and 14, and the relative tumor volume was calculated by using the following formula: *V* = 1/2 × *d* × (*D*)^2^, where *V* is the tumor volume, *D* is the length at the widest point of the tumor, and *d* is the length at the narrowest point of the tumor. At the same time, the weights of the mice were measured by using an analytical balance. The tumor tissue and skin wound were photographed with a ruler by using a camera under anesthesia.

### 2.13 Pathological detection of tumors and assessment of new skin

Solid tumor tissues were fixed in 4% paraformaldehyde, embedded in paraffin, sectioned in paraffin, and sliced into slices with a thickness of about 5 μm, and the tumor tissues were subjected to H&E staining. New skin is stained with H&E.

### 2.14 Tissue reconstruction in chronic wounds

C57 mice (6–7 weeks old, male) were obtained from Shenzhen Rongwan Biotechnology Co., Ltd. Streptozotocin was injected into the abdominal cavity of mice at a dose of 50 mg kg^−1^, once a day for 5 consecutive days. The mice's blood sugar levels were measured by using a blood glucose meter after 14 days. A diabetes model was constructed when blood glucose concentration of mice was greater than 20 mM. The back of the mice was cleaned with depilatory cream, and then a 10 mm diameter skin wound was created on the back of the mice. Diabetic mice were randomly divided into 3 groups (control, hydrogel, and Pd@hydrogel). Different groups of hydrogels were applied to the corresponding wounds and wrapped with gauze.

### 2.15 Wound assessment

The wounds were photographed by using a camera on the 0^th^, 2^nd^, 4^th^, 6^th^, 8^th^, 10^th^, 12^th^, and 14^th^ days, and then ImageJ software was used to measure the area of the skin wound in the picture. The wound area for *x* days is recorded as *A*_*x*_, and then the relative wound area can be calculated by using the following formula:Relative wound area (100%) = *A*_*x*_/*A*_0_ × 100%

### 2.16 Pathological test of new skin

The skin tissue of the back of the mice was harvested for H&E staining, Masson staining, and immunofluorescence to detect CD31.

### 2.17 Statistical analysis

All data were expressed quantitatively as mean ± standard deviation (S.D.). Statistical analysis was conducted by using Microsoft Excel 2013 software. A statistical significance level of the *p* value of 0.05 was selected, with (*) for *p* < 0.05, (**) for *p* < 0.01, and (***) for *p* < 0.001, respectively.

## 3 Results and discussion

### 3.1 Synthesis and characterization of Pd NPs

**Fig. 1:**
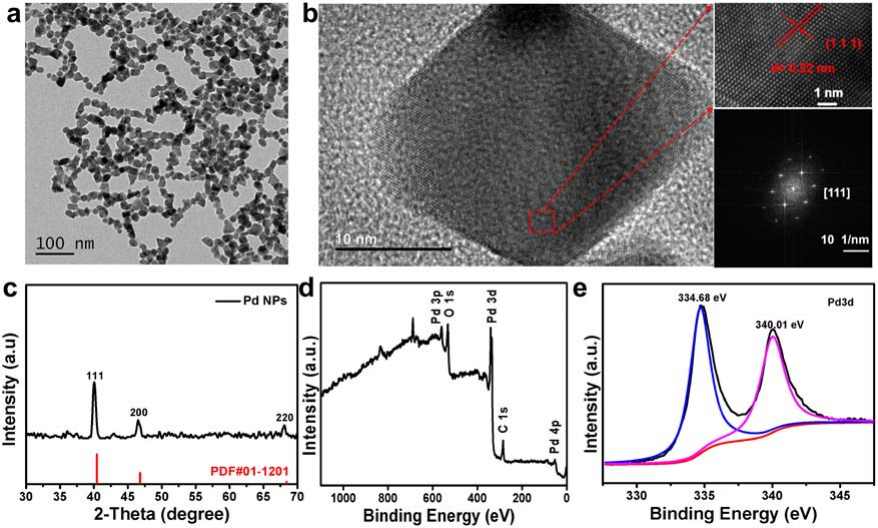
(a) TEM image of Pd NPs. Scale bar, 100 nm. (b) High-resolution TEM image and SAED pattern of Pd NPs. Scale bar, 10 nm (inset, 5 nm). (c) XRD of Pd NPs. (d) Full-range XPS spectrum of Pd NPs. (e) XPS spectrum of Pd elements.

Pd NPs were successfully synthesized by a one-step solvothermal method reported in the literature.^22^ The resulting dark brown Pd NPs were well dispersed in PBS buffer without any aggregates, showing their excellent dispersibility (Fig. S1†). Transmission electron microscopy (TEM) imaging showed that the as-synthesized Pd NPs have a uniform thickness with triangular or quadrilateral shapes (Fig. 1a) of ∼25 ± 5 nm in size. The Pd NPs look like they are connected with each other due to the polymer of PVP coating on the surface of Pd nanoparticles. The hydrodynamic size distribution of the Pd NPs as obtained by dynamic light scattering (DLS) was ∼130 nm due to the surface coating of PVP (Fig. S2†). The high-resolution TEM image and selected area electron diffraction (SAED) pattern showed the atomic lattice (Fig. 1b); despite the different shapes of Pd NPs, adjacent atomic lattices can be seen to be aligned in the same direction, indicating that the Pd NPs have high crystallinity. The measured interplanar spacing was 0.22 nm, corresponding to the (111) lattice plane of the cubic Pd phase. X-ray diffraction (XRD) of the Pd NPs demonstrated crystallinity consistent with the standard peaks (JCPDS card no. 01-1210) without other impurity peaks (Fig. 1c), indicating the purity of the as-prepared samples. The 2*θ* angles observed were 40.4°, 46.7°, and 68.4°, corresponding to the lattice coefficients (111), (200), and (220), respectively. In addition, X-ray photoelectron spectroscopy (XPS) further confirmed the chemical composition of the Pd NPs with peaks observed for elements C, O, and Pd (Fig. 1d). The high resolution XPS spectrum showed two main peaks at 334.6 and 340 eV corresponding to the Pd 3d_5/2_ and Pd 3d_3/2_ orbitals of Pd, respectively, similar to the characteristic of crystalline Pd^0^ as reported in the literature,^23–25^ and which is consistent with the result of XRD. As demonstrated in previous literature,^26^ Pd^0^ can promote the transfer of photoinduced electrons between the conduction bands on the metal surface, thus reducing the recombination of photoinduced carriers, promoting the photothermal conversion and catalytic performance of Pd. These results are highly consistent with those reported previously for fresh Pd NPs,^27,28^ further implying the successful synthesis of pure Pd NPs.

### 3.2 PTT and PDT effects of Pd NPs

**Fig. 2:**
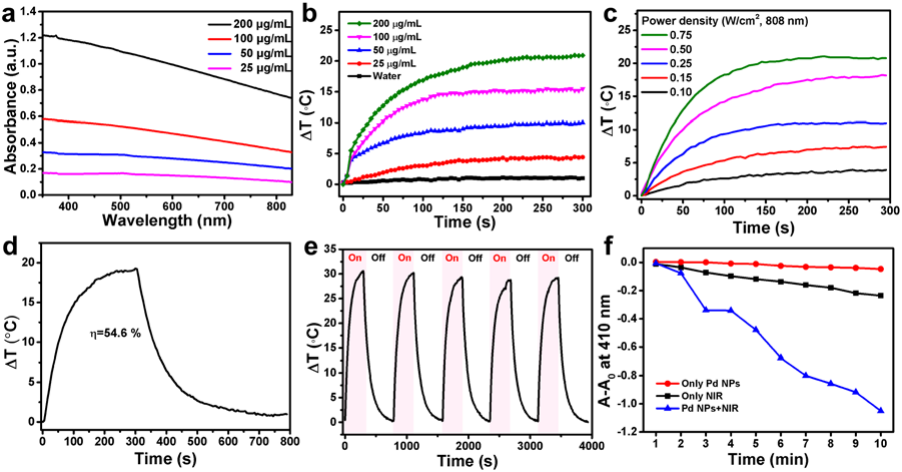
(a) UV-Vis-NIR spectra of Pd NPs in aqueous solution at different concentrations (25–200 μg mL^−1^). (b) Temperature change curve of Pd NPs in aqueous solution at different concentrations upon laser irradiation (808 nm and 0.5 W cm^−2^). (c) Temperature change curve of Pd NPs in aqueous solution (200 μg mL^−1^) with different laser power densities (808 nm). (d) Temperature change curve of Pd NPs in aqueous solution (200 μg mL^−1^) upon laser irradiation (808 nm and 0.5 W cm^−2^) for 300 s and laser shutting off. (e) Photothermal stability of Pd NPs in aqueous solutions (400 μg mL^−1^) irradiated (808 nm and 0.5 W cm^−2^) for 300 s over five cycles. (f) Degradation of DPBF in solution at 410 nm with Pd NPs (200 μg mL^−1^ and 1.0 W cm^−2^).

It is well known that surface plasmon materials, such as Au and Pt NPs, have a strong surface plasmon resonance effect,^29,30^ wherein surface electrons will resonate with light and convert into heat energy under light irradiation.^31^ The efficient photothermal effect depends on the localized surface plasmon resonance to the incident light; thus, the stronger the absorption of materials the higher the induced photothermal capability.^29,32^ UV-Vis-NIR showed that, as the concentration of Pd NPs increases, the greater the absorption in the NIR region. Pd NPs have strong absorption in the NIR region (Fig. 2a), allowing an excellent photothermal effect. Thus, the *in vitro* photothermal performance of the as-synthesized Pd NPs at various concentrations (0, 25, 50, 100, and 200 μg mL^−1^) was evaluated under 808 nm laser irradiation (0.5 W cm^−2^). The temperature elevation of the Pd NP solution was found to be proportional to the concentration (Fig. 2b). For example, at 100 μg mL^−1^, the temperature of the Pd NP solution increased by 15.2 °C after 5 min of laser irradiation; as the concentration increased to 200 μg mL^−1^, the temperature increased by 20.6 °C. In contrast, the temperature of pure water was increased by only 1.8 °C, implying that the 808 nm laser alone is not the cause. On the other hand, the laser power density can also influence the photothermal effect. Therefore, a Pd NP aqueous solution of 200 μg mL^−1^ was irradiated at various laser power densities (0.1, 0.15, 0.25, 0.50, and 0.75 W cm^−2^) to assess the influence on the photothermal effect. The temperature of this Pd NP solution increased as the laser power density increased (Fig. 2c). At a low laser power density of 0.1 W cm^−2^, the temperature increased by only 2.5 °C after 5 min. In contrast, at a laser power density of 0.5 W cm^−2^ and 0.75 W cm^−2^, the temperature of the Pd NP solutions was increased by 17.6 °C and 21 °C, respectively, exhibiting potential PTT applications. Therefore, to further assess the photothermal conversion effect of Pd NPs, the Pd NP solution (200 μg mL^−1^) was irradiated with an 808 nm NIR laser (0.5 W cm^−2^) for 5 min followed by switching-off the laser, producing the typical NIR-induced photothermal heating and cooling curve (Fig. 2d). The temperature of the Pd NP solution rapidly increased to 19.6 °C within 5 min from room temperature, which is high enough to induce cancer cell death by phototherapy. According to a previously reported method,^33^ the photothermal conversion efficiency of Pd NPs is 54.6% (Fig. S3†), which is slightly better compared with that of other metal materials such as Au (23.7%)^34^ and Cu_2−_*x*Se (22%).^35^ Since clinical applications of PTT require long-term exposure to achieve an optimized effect, Pd NPs need to have relatively stable photothermal properties for several hours of laser irradiation to ensure therapeutic outcomes. Therefore, the photothermal stability of the Pd NPs was evaluated through five heating and cooling cycles controlled by laser on and off. The photo-induced heating and cooling profile of the Pd NP solution had minimal change with a negligible decrease after five cycles (Fig. 2e), indicating the excellent photothermal stability of the Pd NPs.

UV-Vis-NIR spectroscopy was also utilized to study the photo-stability of the Pd NPs under laser treatment. After 30 min of 808 nm laser irradiation, the absorption curve of Pd NP solution was almost identical to that of untreated Pd NPs (Fig. S4†), further demonstrating the stability of the synthesized Pd NPs. Therefore, the superior photo-stability of Pd NPs can ensure their excellent photothermal conversion efficiency and photothermal effect as a promising PTA. Several reports have demonstrated that Pd NPs have a strong PTT effect under NIR light irradiation,^36,37^ similar to other surface plasmonic nanomaterials (Au, Pt, and CuS NPs). Combined PDT and PTT can lead to synergistic effects to increase the anticancer performance of materials;^38–40^ nevertheless this has rarely been reported for Pd NPs. Therefore, the combination of PDT and PTT was also explored herein. To evaluate the PDT ability of Pd NPs, 1,3-diphenylisobenzofuran (DPBF), a commonly used probe *in vitro*, was employed to monitor the generation of singlet oxygen (^1^O_2_). The degradation of DPBF causes an absorption decrease at 410 nm, which indicates the amount of ^1^O_2_ generated by the PDT effect.^41^ Pd NPs treated with 808 nm laser illumination could efficiently generate ^1^O_2_, resulting in the decomposition of DPBF with a drop in absorption at 410 nm (Fig. 2f and S5†). The absorption of DPBF for the Pd NP solution decreased significantly over time under 808 nm irradiation, while that of Pd NPs without irradiation and the 808 nm NIR laser without Pd NPs hardly decreased, indicating that Pd NPs can efficiently generate ^1^O_2_ under 808 nm laser irradiation, showing their PDT abilities. Considering that Pd NPs could also generate a remarkable heating effect under 808 nm laser irradiation, the degradation of DPBF due to the photothermal effect was also needed to be clarified in this system. The DPBF solution was heated from 23 °C (ambient temperature) to a specified temperature (33 °C, 43 °C, 53 °C, 63 °C, and 73 °C), and the absorption of DPBF was recorded. Changes in temperature did not lead to a decrease in the absorption of DPBF at ∼410 nm, even at 73 °C. Therefore, Pd NPs can effectively generate ^1^O_2_ to perform PDT under 808 nm laser irradiation.

Firstly, the good biocompatibility of Pd NPs was confirmed in mouse melanoma B_16_F_10_ cells *in vitro* (Fig. S6†). To further verify the PDT effect of Pd NPs, *in vitro* measurements were also performed to evaluate the cellular capacity for ROS generation. B_16_F_10_ cells were cultured with a medium containing 200 μg mL^−1^ Pd NPs, and the H_2_DCFDA probe was utilized to monitor ROS production in the cells. After cellular entrance, the H_2_DCFDA probe could be deacetylated by the intracellular esterase, to produce non-fluorescent deacetylated H_2_DCF, which could then be easily oxidized to fluorescent DCF by ROS such as O_2_˙^−^, ^1^O_2_, and ˙OH.^41,42^ When B_16_F_10_ cells were treated with the H_2_DCFDA probe alone or with 200 μg mL^−1^ of Pd NPs without laser irradiation, minimal green fluorescence was observed, indicating negligible ROS generation. However, when the Pd NP-treated B_16_F_10_ cells were irradiated with an 808 nm NIR laser, the cells produced a significant green intensity, implying that a considerable amount of ROS was generated (Fig. S7†). The green fluorescence brightness of Pd NPs was also similar to the one treated with H_2_O_2_, which further proving that Pd NPs have great potential for PDT applications.

### 3.3 NIR light-regulated drug release

To synthesize a hydrogel with tumor healing and wound repair ability, agarose and chitosan, two commonly used natural polysaccharides with excellent biocompatibility, were utilized as the matrix materials, deacetylated chitosan was dissolved in 1% acetic acid solution for several hours, and then low melting point agarose was added to form a hydrogel at 50 °C. Pd NPs and Pd NP/DOX were loaded onto the hydrogels to form Pd@hydrogel and Pd/DOX@hydrogel, respectively; the hydrogel alone was set as a control named hydrogel. As shown in Fig. S8†, the zeta potential of hydrogel was 32.4 mV, while the zeta potential of Pd@hydrogel and Pd/DOX@hydrogel was 32.2 mV and 36.2 mV, respectively. These hydrogels demonstrated almost similar zeta potentials, suggesting that the Pd NPs and DOX could hardly influence the potentials of hydrogels. In addition, owing to the concerns of hydrogels serving as wound dressings, the stability of hydrogels under different biological conditions was also measure and is presented in Fig. S9†. When the hydrogels were placed in air, there is almost no change in the state of the hydrogels on days 1, 3 and 7, suggesting that these hydrogels can act as excellent wound dressings. When the hydrogels were placed in PBS buffer at different pH values (pH 5.0, 6.5, and 7.4), all hydrogels kept the hydrogel state with excellent stability (Fig. S9†). The hydrogel at all pH values slightly swelled as the hydrogel at the bottom becomes slightly white. The color of Pd@hydrogel and Pd/DOX@hydrogel changed a lot due to the release of Pd NPs and DOX to the upper supernatant. However, there was still a clear boundary between the hydrogel at the bottom and the upper supernatant, revealing that the good stability of the hydrogel under biological conditions.

**Fig. 3:**
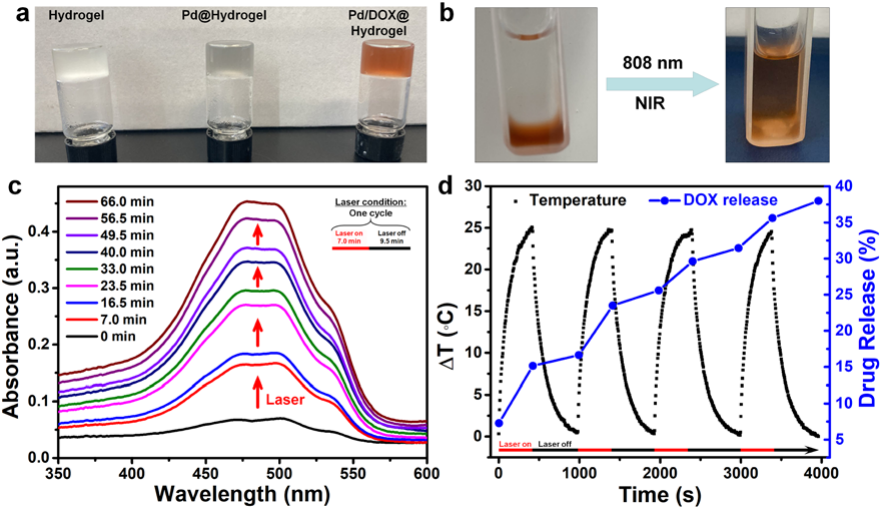
(a) Photo of hydrogels. (b) Photo of Pd/DOX@hydrogel before or after NIR (0.5 W cm^−2^) exposure. (c) Absorbance of DOX (NIR-triggered drug release of Pd/DOX@hydrogel). (d) NIR-triggered drug release of Pd/DOX@hydrogel and the temperature change curve.

With the introduction of Pd NPs and Pd NPs/DOX, the translucent colorless hydrogel developed a gray and brown color, respectively (Fig. 3a). DOX in Pd/DOX@hydrogel was not released in PBS buffer without treatment, because the hydrogel is a thermo-sensitive hydrogel, which can melt and release drug molecules only when it exceeds a critical value (45 °C). In comparison, Pd/DOX@hydrogel was irradiated under an 808 nm laser, and the DOX can be easily released from Pd/DOX@hydrogel due to the photothermal effect, causing the solution color change from colorless to brown (Fig. 3b), revealing that the DOX was released from Pd/DOX@hydrogel in a NIR light-triggered release manner for precise cancer therapy. To further evaluate the photo-induced release behavior, Pd/DOX@hydrogel was irradiated with 808 nm laser “on–off” and the concentration of DOX in the supernatant was measured by UV-Vis-NIR spectroscopy. Pd/DOX@hydrogel was treated with the 808 nm laser on (7.0 min) and off (9.5 min) for four cycles, and the absorption of supernatant PBS buffer was measured to monitor the DOX release (Fig. 3c). For each cycle, the absorption was significantly increased after 808 nm laser stimulation, whereas that of the laser off for 9.5 min showed only a slight increase (Fig. 3d). Additionally, the temperature increased by 25 °C with minimal variation for each cycle. After four cycles of laser treatment, the ultimate release rate of DOX was achieved at ∼37.6%. Thus, drug release was great related to the photothermal-induced temperature increase, leading to a photo-triggered drug release mechanism. Therefore, the photothermal agents and anticancer drug-loaded Pd/DOX@hydrogel can serve as combined PTT and photo-triggered chemotherapy with a synergistic effect. To explore the drug release effect of the tumor microenvironment, 0.2 mL of Pd/DOX@hydrogel were placed into PBS buffer solutions at different pH (5.0, 6.5, and 7.4); the absorbance of the supernatant was highest at pH 5, indicating that the slightly acidic environment can promote the release of DOX (Fig. S10†).

### 3.4 Characterization of hydrogels

**Fig. 4:**
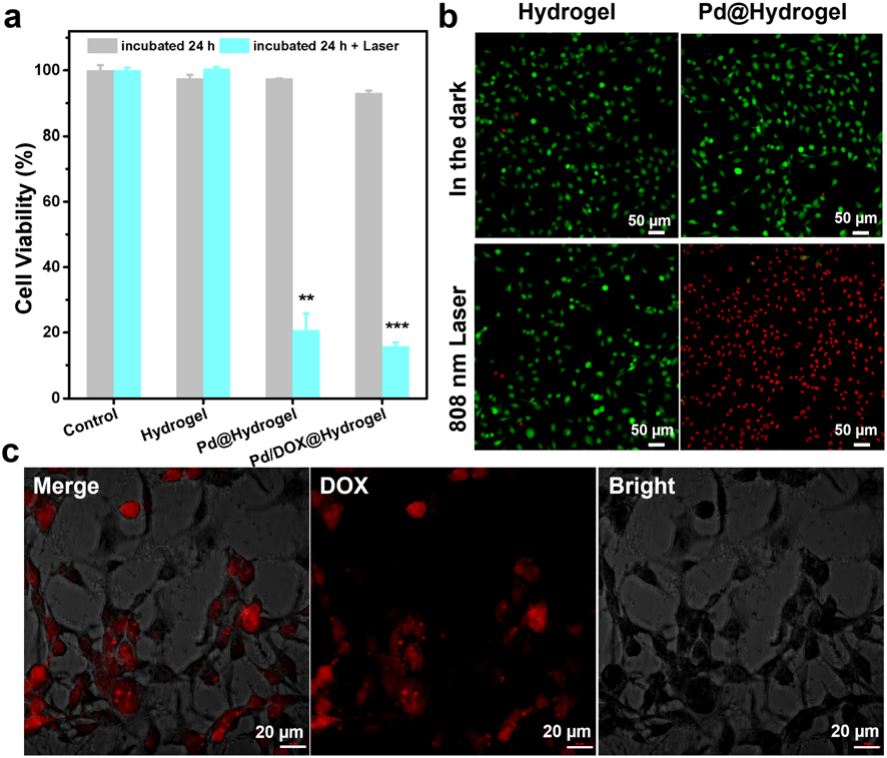
(a) Cytotoxicity of B_16_F_10_ cells treated for 24 h with hydrogels with or without irradiation (laser 808 nm, 0.5 W cm^−2^) (**, *p* < 0.01; ***, *p* < 0.001). (b) CLSM images of calcein AM/propidium iodide-stained B_16_F_10_ cells after the different treatments (hydrogel and Pd@hydrogel, 200 μg mL^−1^; power density of 808 nm laser, 0.5 W cm^−2^). (c) CLSM of B_16_F_10_ cells treated with Pd/DOX@hydrogel under 808 nm laser irradiation (0.5 W cm^−2^).

A hydrogel is composed of a polymer material with a three-dimensional network structure and high moisture content. Therefore, the physical and chemical properties of hydrogels are similar to those of soft tissue *in vivo*.^43^ Hydrogels are frequently utilized in the biomedical field due to their good biodegradability, low immunogenicity, anti-oxidation abilities, and cell adhesion. A variety of natural materials, including collagen, alginate, gelatin, hyaluronic acid, chitosan, and silk fibroin, have been widely developed in the development of hydrogels. Herein, the hydrogel matrix was composed of agarose and chitosan, which have been widely used as wound antiseptic dressings and drug-loading gels to achieve sustained drug release.^44^ Pd/DOX@hydrogel can efficiently convert NIR light into thermal energy, resulting in both a photothermal effect and photothermally induced drug release. Thus, it can instantly kill residual tumor cells by PTT and chemotherapy. In addition, the wound healing ability of hydrogels was investigated due to the antibacterial effect in postoperative infection. The three types of hydrogels (that is hydrogel control, Pd@hydrogel, and Pd/DOX@hydrogel) were evaluated. The toxicity of hydrogels with and without 808 nm laser treatment was explored using B_16_F_10_ cells co-cultured with hydrogels and treated under different conditions. CCK-8 assay was used to evaluate the cell viability. All hydrogels in the dark showed negligible toxicity, with a cell survival rate of more than 90% (Fig. 4a), demonstrating the superb biocompatibility of hydrogels. In addition, we co-cultured all the hydrogels with HDF cells for several days; the cell survival rate of the hydrogel alone was the greatest on day 5, indicating that the composite hydrogels have good biocompatibility (Fig. S11†).

To explore the photothermal effect of Pd@hydrogel, 200 μg mL^−1^ Pd NPs were uniformly dispersed in the hydrogel at 60 °C and allowed to cool to room temperature. Similar to that of Pd NP solution, the temperature of Pd@hydrogel (200 μg mL^−1^) rapidly increased from 27 °C to 59 °C under an 808 nm laser for 5 min (Fig. S12†), with a photothermal conversion efficiency as high as 32.79%. To explore its photothermal stability, the temperature of Pd@hydrogel treated with the 808 nm laser switching on and off over four cycles was characterized. The temperature still reached the high enough photo-induced temperature, suggesting that the Pd NPs retain an excellent photothermal stability in Pd@hydrogel. For Pd@hydrogel with 200 μg mL^−1^ Pd NP loading, the B_16_F_10_ cells could be largely killed under 808 nm laser irradiation owing to the photothermal effect, exhibiting the efficient PTT anticancer ability of Pd NPs. Similar results were also obtained with live/dead cell staining with calcein AM and propidium iodide. Pd@hydrogel without the laser and the laser alone didn't induce negligible cell death, while Pd@hydrogel treated with 808 nm laser irradiation significantly killed most B_16_F_10_ cells as shown by the red fluorescence in the confocal image (Fig. 4b). In addition, Pd@hydrogel also generated ROS in B_16_F_10_ cells (Fig. S13†), demonstrating its effective photodynamic effect.

Furthermore, when 200 μg mL^−1^ Pd NPs and 200 μg mL^−1^ DOX were simultaneously introduced into Pd/DOX@hydrogel, the viability of B_16_F_10_ cells treated with Pd/DOX@hydrogel and 808 nm laser irradiation was also greatly reduced to less than 20%, mainly by the PTT effect and photothermally induced DOX release. Pd/DOX@hydrogel generated local heat under NIR light irradiation due to the photothermal effect of Pd NPs, and the thermosensitive hydrogel became soft at ∼45–50 °C with a faster release of the encapsulated DOX. The photo-induced DOX release from Pd/DOX@hydrogel led to further killing of B_16_F_10_ cells in a photo-controlled manner. Therefore, the *in vitro* results further proved that Pd/DOX@hydrogel can undergo efficient PTT and photothermally triggered chemotherapy to treat cancer cells. To further verify the NIR-triggered DOX release, B_16_F_10_ cells were treated with Pd/DOX@hydrogel and the cellular fluorescence of DOX was observed by CLSM after 808 nm laser irradiation. The strong red fluorescence indicated the successful entrance and distribution of DOX in B_16_F_10_ cells, further proving that NIR can trigger the cellular release of drugs (Fig. 4c).

### 3.5 *In vivo* tumor recurrence inhibition of hydrogels and skin repair ability

**Fig. 5:**
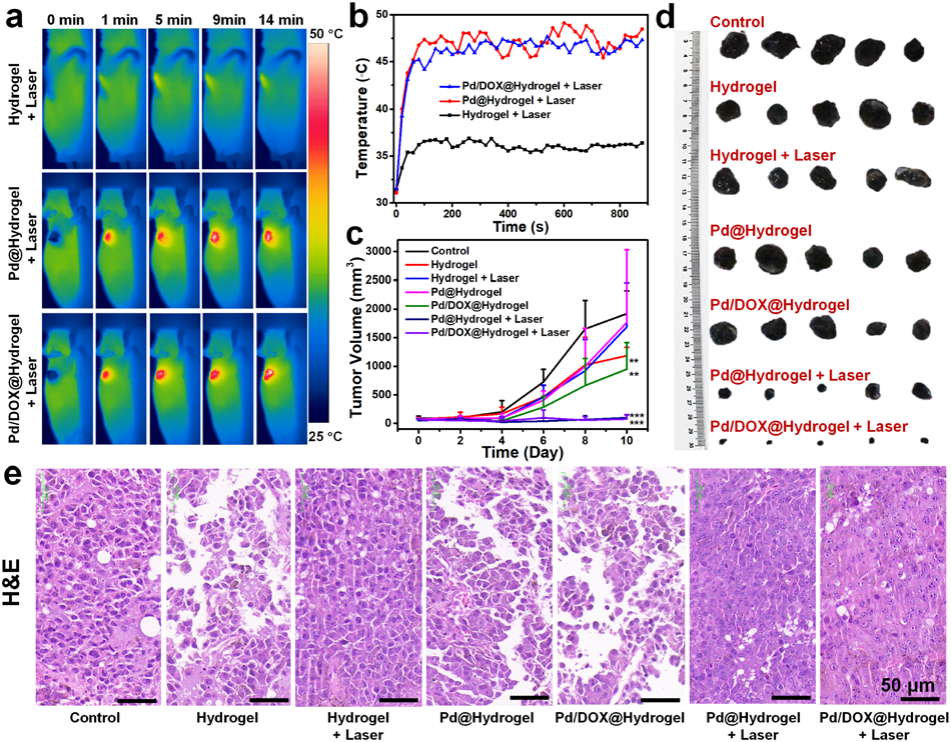
(a) Infrared thermal photo at different time intervals of B_16_F_10_ tumor-bearing mice in which 50 μL of hydrogel were applied *in situ* under 808 nm laser irradiation. The laser power density was 0.5 W cm^−2^. (b) Temperature *versus* irradiation time curve of B_16_F_10_ tumor-bearing mice. (c) Tumor volume of mice from different groups (**, *p* < 0.01; ***, *p* < 0.001). (d) Excised tumors from B_16_F_10_ tumor-bearing mice after different treatments (10 days). (e) H&E-stained with tumor after different treatments. Scale bar: 50 μm.

Owing to the effectiveness of Pd/DOX@hydrogel to kill cancer cells *in vitro*, the *in vivo* antitumor performance was further assessed to evaluate the therapeutic effect of hydrogels in the treatment of tumors after surgery, the potential of postoperative therapy, and the ability to inhibit tumor recurrence. B_16_F_10_ tumor models were constructed by subcutaneous injection of 5 × 10^5^ cells in 100 μL of DMEM into the flank region of the right front of mice. A 10 mm diameter skin wound was created on the upper part of the tumor when the diameter of the melanoma reached about 4 mm. But the tumor tissue is not completely cut off, simulating the setting of tumor surgical resection. Mice were randomly divided into seven groups with different treatments: (i) control; (ii) hydrogel; (iii) Pd@hydrogel; (iv) hydrogel + laser; (v) Pd/DOX@hydrogel; (vi) Pd@hydrogel + laser; (vii) Pd/DOX@hydrogel + laser. To investigate the photothermal properties of the hydrogels, the temperature of the wound treated with hydrogel, Pd@hydrogel, and Pd/DOX@hydrogel with 808 nm laser irradiation was recorded by infrared thermography (FLIR A300, USA) (Fig. 5a). For the hydrogel group irradiated with an 808 nm laser, there was only a slight temperature increase to ∼36–37 °C (Fig. 5b), similar to the body temperature of mice without photothermal therapy. In distinct contrast, the Pd@hydrogel and Pd/DOX@hydrogel groups treated with an 808 nm laser demonstrated efficient photothermal capability and a rapid temperature increase to ∼46–48 °C (Fig. 5b), significantly causing the cell death and inducing thermally triggered drug release. Therefore, postoperative antitumor therapy was performed to evaluate the phototherapy and photo-induced drug delivery. The body weight and tumor volume of the mice were monitored every 2 days to monitor the safety and tumor inhibition ability of mice. All mice were monitored for 10 days, with tumor volumes in some mice reaching over 3000 mm^3^, at which time the experiment was stopped due to the health concern and the guidelines for the care and use of laboratory mice. The body weight of mice in all treated groups did not change significantly after treatment (Fig. S14†), revealing that the hydrogels led to minimal toxicity *in vivo*. Furthermore, the control, hydrogel, hydrogel with laser, and Pd@hydrogel groups showed negligible tumor inhibition, with similar tumor growth curves (Fig. 5c) and minimal tumor suppression. In comparison, the Pd/DOX@hydrogel without laser irradiation group showed better tumor inhibition likely due to the release of DOX within the wound area. In distinct contrast, the tumors treated with Pd@hydrogel and Pd/DOX@hydrogel with 808 nm laser irradiation were efficiently suppressed, with the tumor almost disappearing (Fig. 5c). Thus, Pd@hydrogel can serve as a combined PTT and PDT platform with synergistic antitumor effectiveness. Furthermore, it demonstrated even better antitumor capability in comparison to Pd@hydrogel due to the combined PTT, PDT, and photothermally induced chemotherapy (Fig. 5c and d). The excellent *in vivo* postoperative antitumor effectiveness of Pd/DOX@hydrogel with infrared imaging ability provides a promising therapeutic mode for tumor treatment.

To further explore the inhibitory effect of hydrogels on melanoma tumors, the tumor tissues of each group were excised for hematoxylin and eosin (H&E) staining. The tumor cells treated with the control, hydrogel, hydrogel with laser irradiation, and Pd@hydrogel groups were nearly intact (Fig. 5e), indicating that the tumor cells were not affected by these treatments, consistent with the above results. In contrast, Pd/DOX@hydrogel treatment induced tumor cell damage to some degree, ascribed to the minimal release of DOX. Finally, Pd@hydrogel and Pd/DOX@hydrogel with 808 nm laser irradiation significantly damaged the tumor tissue with most of the cells damaged as observed by the blue signal of cell nuclei stained with hematoxylin, which were shapeless compared with the cells of the control group (Fig. 5e). The tumor cell killing ability of Pd@hydrogel and Pd/DOX@hydrogel was respectively attributed to PTT/PDT and PTT/PDT with photo-induced chemotherapy. These results further show that Pd/DOX@hydrogel exploits combined PTT, PDT, and photo-triggered chemotherapy with efficient inhibitory effects on tumors.

Hydrogels are promising wound healing materials. Thus, to explore the wound repair effect of hydrogels on wounds, wound areas were photographed periodically after the different treatments. The wounds of mice treated with the above hydrogels were significantly repaired after 10 days (Fig. S15†). Usually, the larger the tumor volumes, the larger the wound areas for all groups, which means that the tumor inhibition ability would be closely related to that of skin healing outcomes. For all treated groups, good skin healing ability was observed compared with the control group. However, the laser-treated groups (hydrogel + laser, Pd@hydrogel + laser, and Pd/DOX@hydrogel + laser) exhibited better wound healing ability; thus, the laser can influence skin healing probably through the killing of bacteria in the wound area. The Pd@hydrogel and Pd/DOX@hydrogel groups treated with a laser also demonstrated excellent wound healing capability (Fig. S16†). In addition, the PTT effect can also effectively inhibit bacteria and further promote wound repair, similar to daptomycin micelle-stabilized palladium nanoflowers.^45^ Overall, it is likely that the treatment of hydrogels can stimulate the growth of endothelial cells, promote the growth of new blood vessels on the wound surface, and inhibit the recurrence of inflammation, thus further promoting wound healing.

And some mice formed a wound-induced callus, such as those in the Pd@hydrogel with laser treatment group (Fig. S16†), and thus the wound photos may not exactly reflect wound healing performance and the healing ability needs to be further investigated. To clearly study the state of cells and the distribution of blood vessels, the new skin of mice in each group was collected and stained with H&E. The control group had more cancer cells compared with the other treatment groups (Fig. S16†) and the cancer cells invaded adjacent skin tissue, since the tumor tissue continued to grow beneath the skin. By comparison, Pd@hydrogel and Pd/DOX@hydrogel groups with laser treatment had fewer cancer cells. Thus, Pd@hydrogel and Pd/DOX@hydrogel have a good therapeutic effect on cancer cells under NIR irradiation, similar to that of tumor volume inhibition as described above (Fig. 5c and d). In addition, new blood vessel formation was observed with all hydrogel treatments with visible new skin growth (green area, first row of Fig. S16†); thus, all hydrogels can promote wound healing during postoperative therapy. Therefore, Pd/DOX@hydrogel demonstrated an excellent photothermal and photodynamic effect as well as photo-triggered chemotherapy both *in vitro* and *in vivo*, with promising potential for clinical translation. To further illustrate the safety of hydrogels, the major organs of each group were stained with H&E. All organs from these hydrogels demonstrated similar results compared with the control group (Fig. S17†). It is suggested that Pd @hydrogel and Pd/DOX@hydrogel could cause negligible damage or inflammatory lesions in the major organs of tumor-bearing mice 10 days after the PTT. Those results should be attributed to the fact that the Pd NPs and DOX could be released to the wound and tumor sites, but hardly circulate to other major organs.

### 3.6 Wound healing ability for a chronic wound model *in vivo*

As Pd@hydrogel demonstrated admirable antitumor ability as well as skin healing capability, in order to further explore the potential wound healing ability of hydrogels, a chronic wound healing model was constructed for evaluation. As diabetic animal models have been widely used in chronic wound healing research because it is hard to heal, some researchers take it as the target broadly.^46,47^ To investigate the potential in patients with diabetes, the wound healing ability of hydrogels to cure a diabetic wound is explored. Herein, Pd@hydrogel without DOX loading was utilized to study the healing ability for diabetic wounds without tumor bearing. Keeping the wound moist for chronic wounds can promote recovery, improve the wound healing rate, and reduce the occurrence of complications.^48^ Hydrogel dressings not only keep the wound moist and remove excess exudate but are also impermeable to microorganisms and allow gas exchange for wound healing.^49^

**Fig. 6:**
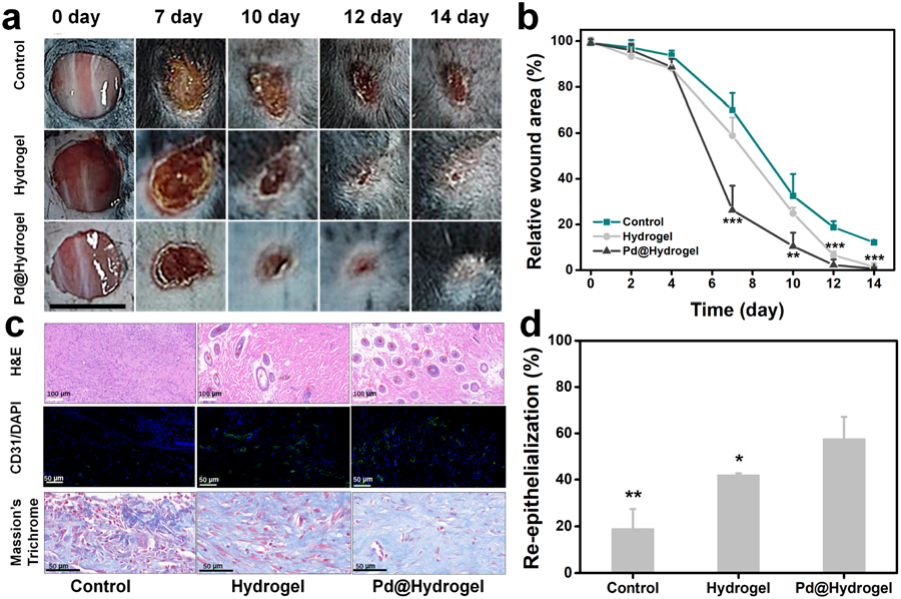
Tissue reconstruction of chronic wounds. (a) Representative photos of the diabetic wound after different treatments after 14 days. (b) Relative wound area of the diabetic wound after different treatments. (c) Photographs of H&E staining, CD31/DAPI staining, and Masson's trichrome staining. (d) Quantitative analysis of CD31-positive vessels (*, *p* < 0.05; **, *p* < 0.01; ***, *p* < 0.001).

Hyaluronic acid and carboxymethyl chitosan, as two of the commonly employed natural polymer materials, are widely harnessed in the research of bioengineered skin substitutes, bioscaffolds, drug carriers, and biological dressings. Here, a mouse model with a diabetic wound was established with subsequent treatments (control, hydrogel, and Pd@hydrogel), and the wound healing status was monitored accordingly. The wound healing effect of the hydrogel and Pd@hydrogel groups was significantly better than that of the control group (Fig. 6a). More importantly, after 14 days, the diabetic wounds in the hydrogel and Pd@hydrogel groups were almost completely healed, while the diabetic wounds in the control group were only healed up to 18.5% of the initial wound area (Fig. 6b). Thus, Pd@hydrogel had a good effect on the healing of this type of chronic wound. To further study the mechanism of Pd@hydrogel in postoperative tissue reconstruction and skin repair, the healing ability was then evaluated. H&E staining of wound tissue after 14 days revealed the healing process of wounds in different treatment groups at the histological level. The chronic wounds were significantly healed (Fig. 6c), with new blood vessel formation as promoted by the hydrogel. Chitosan in Pd@hydrogel scaffolds upregulated vascular endothelial growth factor (VEGF) expression, which in turn stimulated angiogenesis and may be responsible for improved new capillary formation.^50^ Immunofluorescence and Masson's trichrome staining were employed to evaluate angiogenesis and epithelialization, respectively. Platelet-endothelial cell adhesion molecule 1 (CD31) is considered a marker of endothelial cells, suggesting capillary formation.^51^ Therefore, to investigate the effect of the hydrogel on angiogenesis, the expression of CD31 was assessed at the wound site. Compared with the control group, wound scaffolds had significantly higher CD31-positive blood vessel density following treatment with hydrogels, consistent with the quantitative results of CD31-positive cells. Meanwhile, Masson's trichrome staining was utilized to infer the state of collagen deposition in the wound. More collagen fibers covered the hydrogel scaffolds in wounds in comparison to the control group. After 14 days, the degree of collagen degeneration eased in the control group, and the hydrogel and Pd@hydrogel groups had more neovascularization, with a clearly demarcated dermis. Quantitative results also confirmed the improvement of re-epithelialization by hydrogel and Pd@hydrogel scaffolds (Fig. 6d). No significant changes or differences were observed in the body weight of all groups of mice after the different treatments, indicating the low toxicity of Pd@hydrogel (Fig. S18†). Thus, both the control hydrogel and Pd@hydrogel can promote the formation of new blood vessels and the growth of collagen fibers, thereby promoting wound healing. Agarose and chitosan-based hydrogels therefore demonstrated excellent wound healing ability and are a promising platform for postoperative therapy with superior treatment performance.

Until now, there are still significant challenges in the clinical application of nanoparticle injection or transdermal delivery for PTT, such as particle aggregation and the potential toxicity of excessive mental ions, which are not conducive to tissue regeneration.^52–54^ In addition, in clinical applications, it is difficult to inject PTAs into the tumor after tumor resection for PTT and chemotherapy. Multifunctional wound dressings can provide a simple and effective strategy to overcome these challenges. Pd/DOX@hydrogel, as a novel multifunctional hydrogel platform, showed outstanding superiority to others in tumor recurrence and wound healing. Firstly, photo-responsive release behavior can be obtained. Pd/DOX@hydrogel was fabricated by using a low-melting point hydrogel based on agarose, which softens at 40–45 °C. Under 808 nm laser irradiation, the temperature of Pd/DOX@hydrogel could increase to about 45 °C, and the DOX release of Pd/DOX@hydrogel could be further increased accordingly, resulting in achieving the photo-triggered effect of cancer drug therapy. Secondly, pH-responsive release behavior can also be achieved. The acidic microenvironment of tumor can promote DOX release from Pd/DOX@hydrogel, and thus it can play a positive role in inhibiting tumor recurrence. Thirdly, the hydrogel matrix is composed of FDA-approved natural polymers of agarose and chitosan, which can be used as both wound antiseptic dressings and drug-loading reservoirs to achieve sustained drug release for cancer therapy.

## 4 Conclusions

In conclusion, a biocompatible Pd NP system was successfully synthesized by a sample solvothermal method, which demonstrated an efficient photothermal effect with excellent photostability and photodynamic effects simultaneously under 808 nm NIR irradiation. The as-synthesized Pd NPs together with hydrophilic DOX were employed to construct Pd/DOX@hydrogel based on two natural polymers agarose and chitosan, with superior biocompatibility. Pd/DOX@hydrogel exhibited effective photothermal and photodynamic effects both *in vitro* and *in vivo*, and can be coated on postoperative wounds after tumor resection to achieve PTT and PTD with a synergistic effect. In addition, the photothermal effect of Pd/DOX@hydrogel can heat the hydrogel, leading to photo-induced drug release in a controlled manner. Pd/DOX@hydrogel showed significant tumor eradicating ability for residual tumors by combined PTT/PDT and photo-triggered chemotherapy as well as a potential wound healing effect. Therefore, this strategy not only has potential for the biological application of Pd NPs in the adjuvant therapy of solid tumor surgery but also has potential applications for Pd NPs in future research in other biomedical areas.

## Ethical statement

All healthy male BALB/c-nude mice (6–8 weeks old) were purchased from Zhuhai Baishitong Biotechnology Co., Ltd, and maintained under pathogen-free conditions at the SPF Laboratory Animal Center of Shenzhen Rongwan Biotechnology Co., Ltd. All *in vivo* experiments followed the protocol approved by the Animal Care and Use Committee of SPF Laboratory Animal Center of Shenzhen Rongwan Biotechnology Co., Ltd. The license number of the laboratory animal is SYXK 2022-0292. In addition, all animal procedures were performed in accordance with the Guidelines for Care and Use of Laboratory Animals of Shenzhen Technology University, and approved by the Animal Ethics Committee of Shenzhen Technology University.

## Author contributions

X. Yin synthesized and characterized the nanomaterials, and conducted cell and animal experiments. F. Ai supervised the project and wrote the manuscript. T. Fan and N. Zheng participated in the animal test. L. Yan, J. Yang and S. He directed the material synthesis and analysis data. F. Ai and J. Hu supervised the project and revised the manuscript. All authors have read and agreed to the published version of the manuscript.

## Conflicts of interest

All authors declare no conflict of interest.

## Supplementary Material

NA-005-D2NA00897A-s001
