# Supplementary material for: Palladium nanoparticle based smart hydrogels for NIR light-triggered photothermal/photodynamic therapy and drug release with wound healing capability
Source: Nanoscale Adv. 2023 Feb 14;5(6):1729–39. doi: 10.1039/d2na00897a (PMC10012852; doi:10.1039/d2na00897a)
Supplement: NA-005-D2NA00897A-s001 [file NA-005-D2NA00897A-s001.pdf]

Electronic Supplementary Material (ESI) for Nanoscale Advances.
This journal is © The Royal Society of Chemistry 2023

Supporting Information

Palladium nanoparticles based smart hydrogels for NIR light-triggered photothermal/photodynamic therapy and drug release with wound healing capability

Xiuzhao Yin,[a] Taojian Fan,[a] Nannan Zheng,[a] Jing Yang,[b] Li Yan,[b,c] Shuqing He,[b,c] Fujin Ai,*[b,c] Junqing Hu[a,b,c]

[a] College of Applied Technology, Shenzhen University, Shenzhen 518060, P. R. China.

[b] College of Health Science and Environmental Engineering, Shenzhen Technology University, Shenzhen 518118, P. R. China

[c] Shenzhen Bay Laboratory, Shenzhen 518132, P. R. China

Email: aifujin@sztu.edu.cn

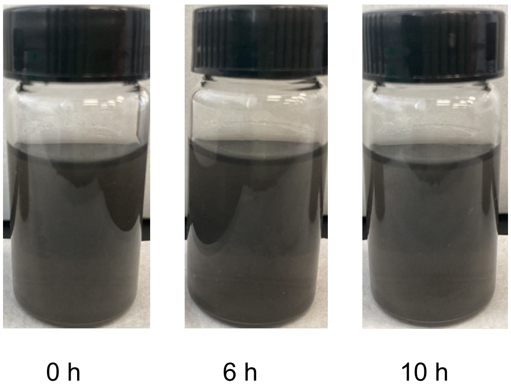

Fig S1. Photographs of Pd NPs in PBS solution at different times.

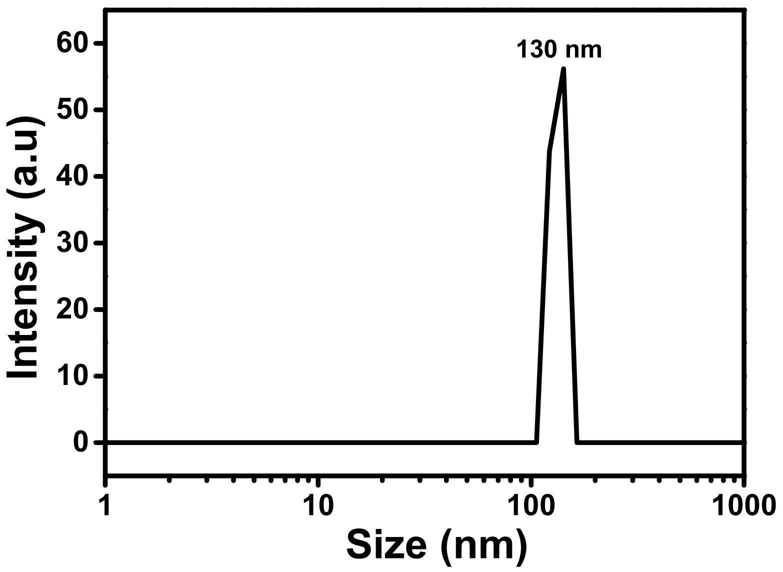

Fig S2. Size distribution of the Pd NPs in PBS aqueous solution.

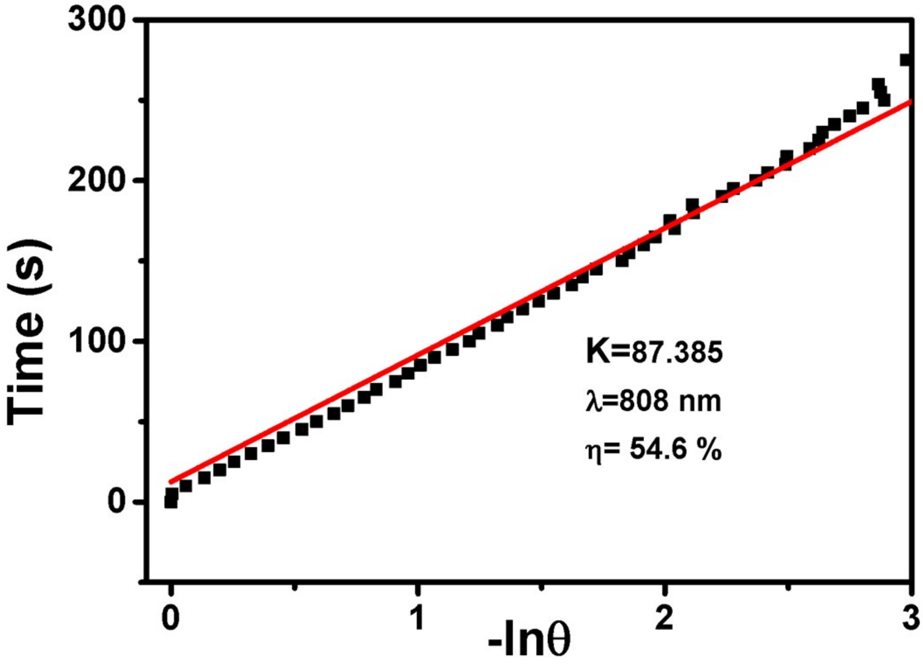

Fig S3. The system heat transfer time constant is calculated using the linear time data of panel cooling.

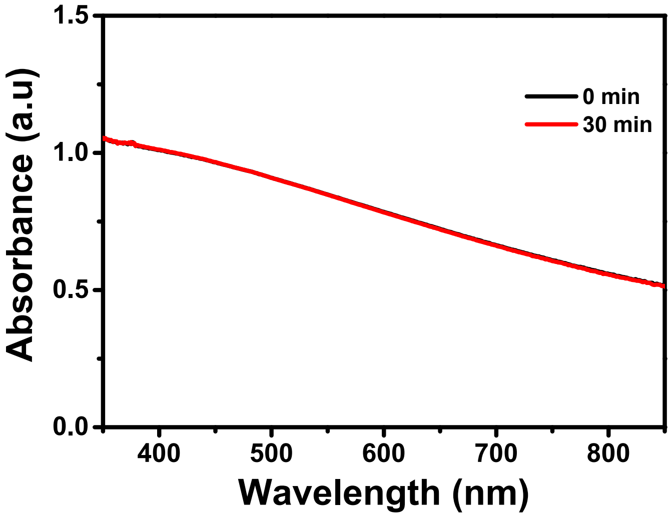

Fig S4. The absorption spectrum of Pd NPs before and after laser irradiation for 30 min.

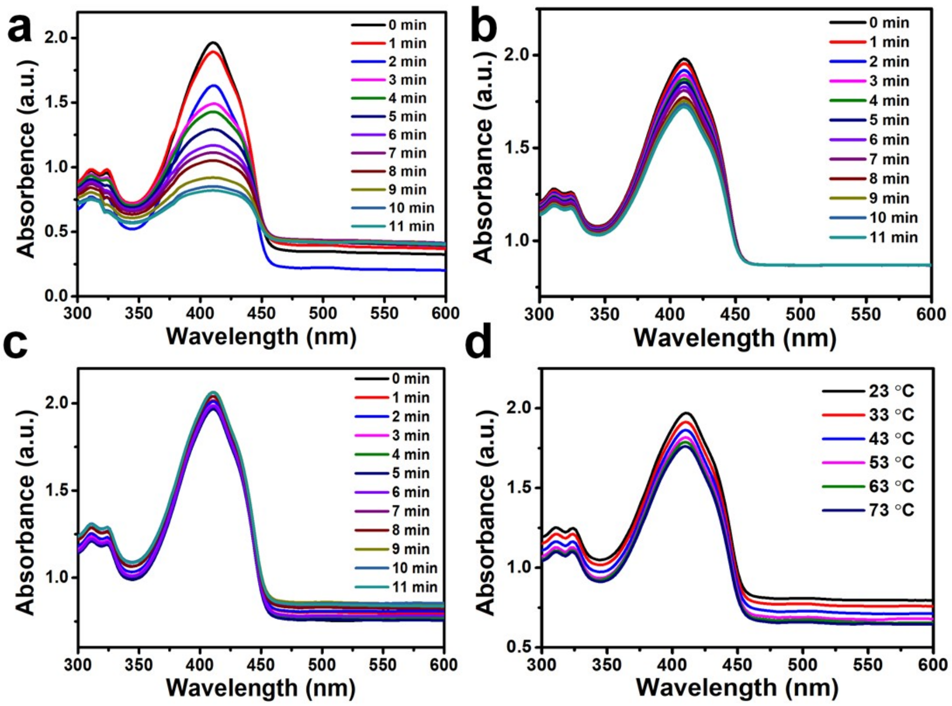

Fig S5. a) The absorbance of DPBF in solution with Pd NPs under 808 nm laser irradiation (200 μg/mL, 1 W/cm^2). b) The absorbance of DPBF in solution with Pd NPs (200 μg/mL). c) The absorbance of DPBF in solution under 808 nm laser irradiation (1 W/cm^2). d) The absorbance of DPBF in solution at different temperature (23 ºC, 33 ºC, 43 ºC, 53 ºC, 63 ºC and 73 ºC)

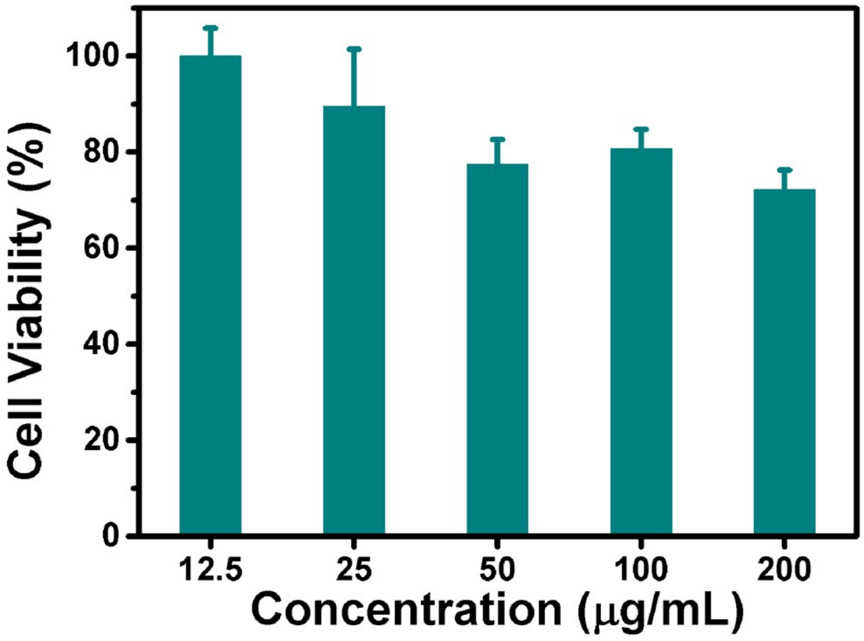

Fig S6. Cell Viability of $B_{16}F_{10}$ cells after different concentration of Pd NPs.

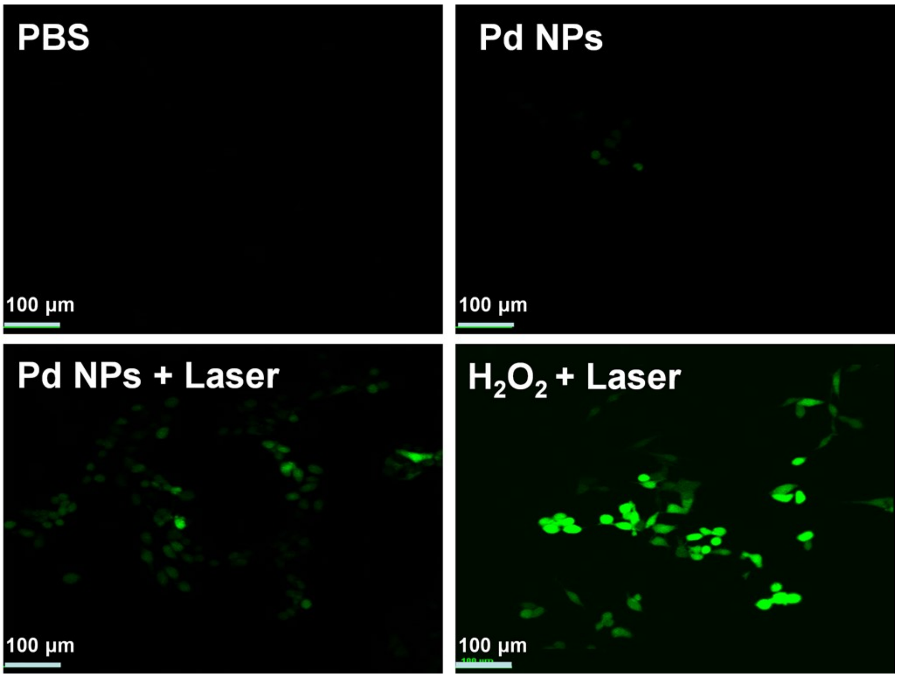

Fig S7. CLSM images of H_2DCFDA-stained $B_{16}F_{10}$ cells after different treatments, scale bar, 100 μm.

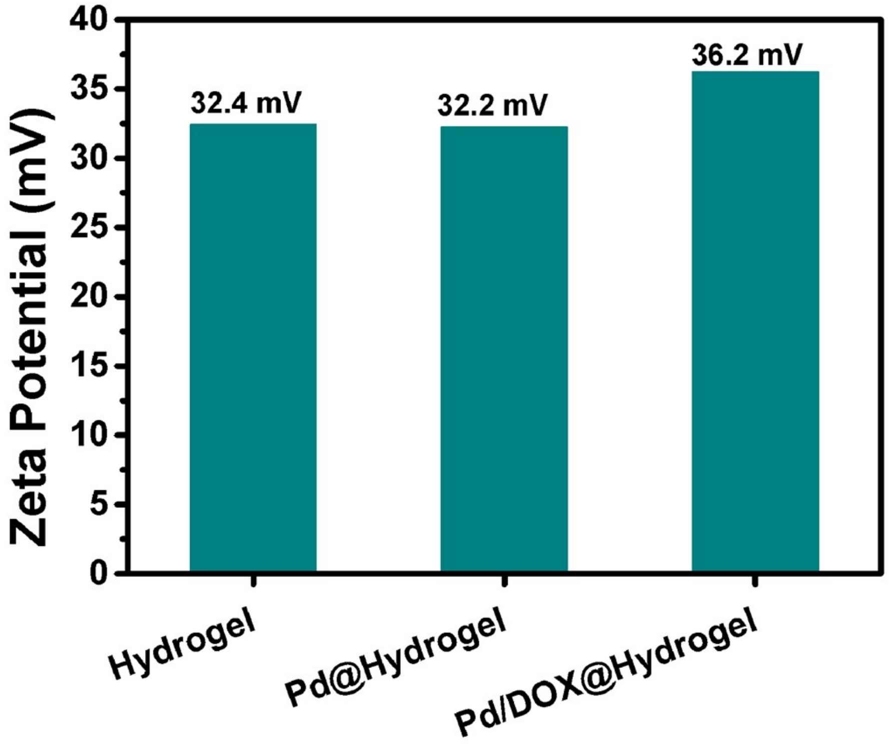

Fig. S8 The zeta potential of Hydrogel, Pd@Hydrogel and Pd/DOX@Hydrogel.

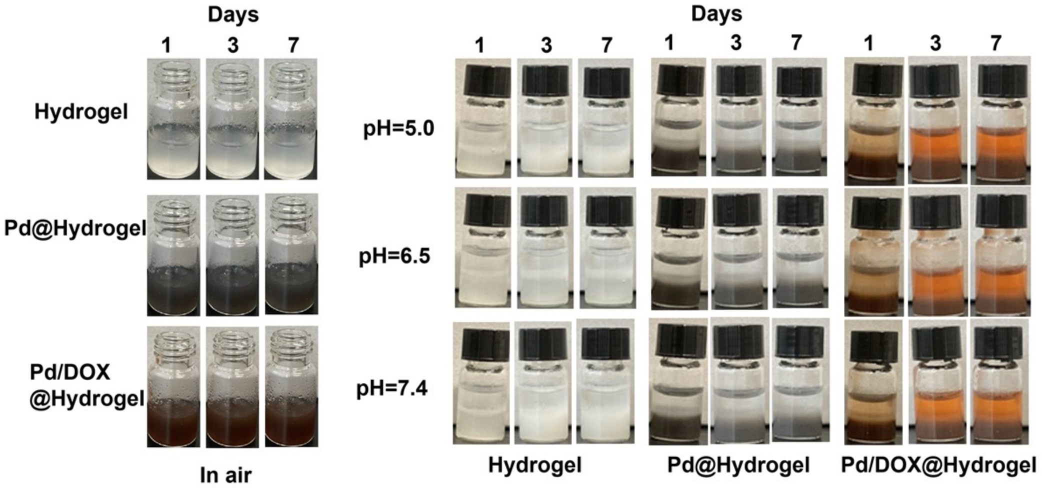

Fig. S9 Digital photos of the hydrogels (Hydrogel, Pd@Hydrogel and Pd/DOX@Hydrogel) alone in air (left) and in PBS buffers at different pH values (right) at 1, 3 and 7 days.

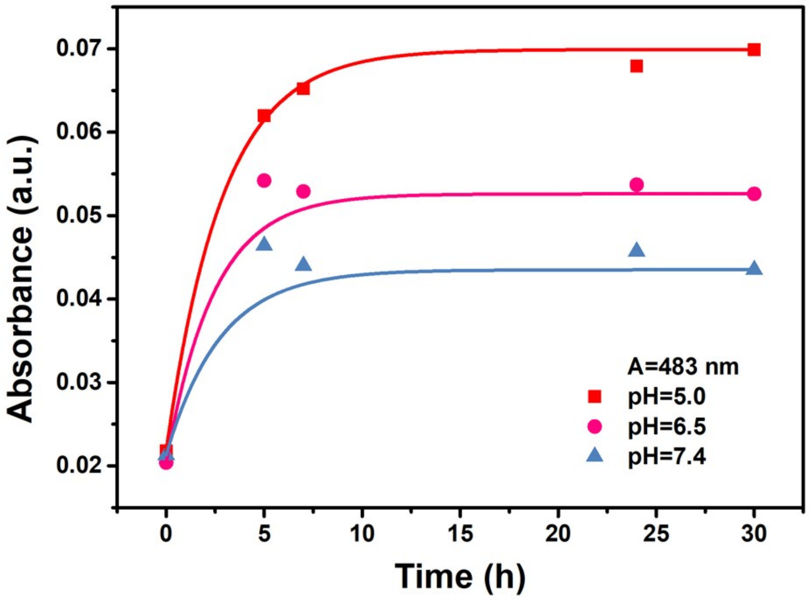

Fig S10. The absorbance of supernatant of Pd/DOX@Hydrogel with different pH buffer.

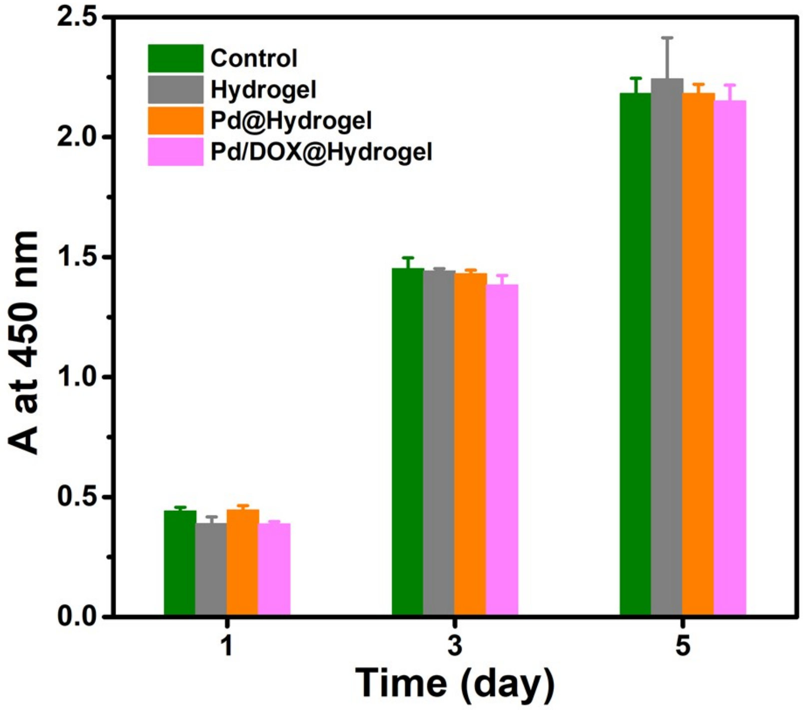

Fig S11. Cell proliferation of HDFs seeded on Hydrogel, Pd@Hydrogel, and Pd/DOX@ Hydrogel for 1, 3, and 5 days.

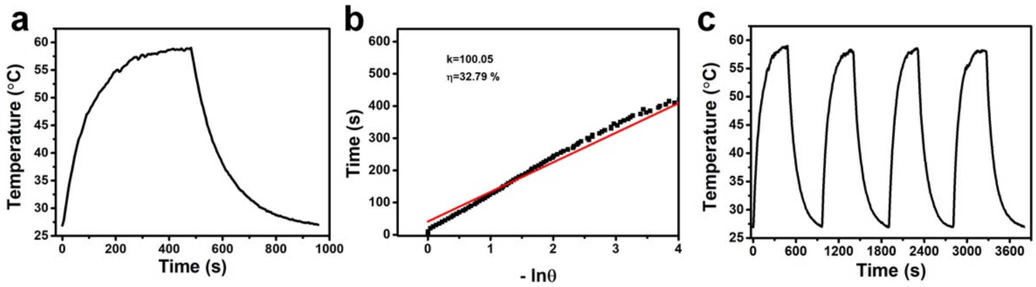

Fig S12. a) Temperature change curve of Pd@Hydrogel (200 μg/mL) upon Laser (808 nm, 0.5 W/cm^2) and Laser shutting shown. b) The system heat transfer time constant is calculated using the linear time data of panel cooling. c) The absorbance of DPBF in solution under 808 nm laser irradiation (1 W/cm^2). d) Photothermal stability of Pd@Hydrogel in aqueous solutions (200 μg/mL) irradiated (808 nm, 0.5 W /cm^2) for 300 s in 4 cycles.

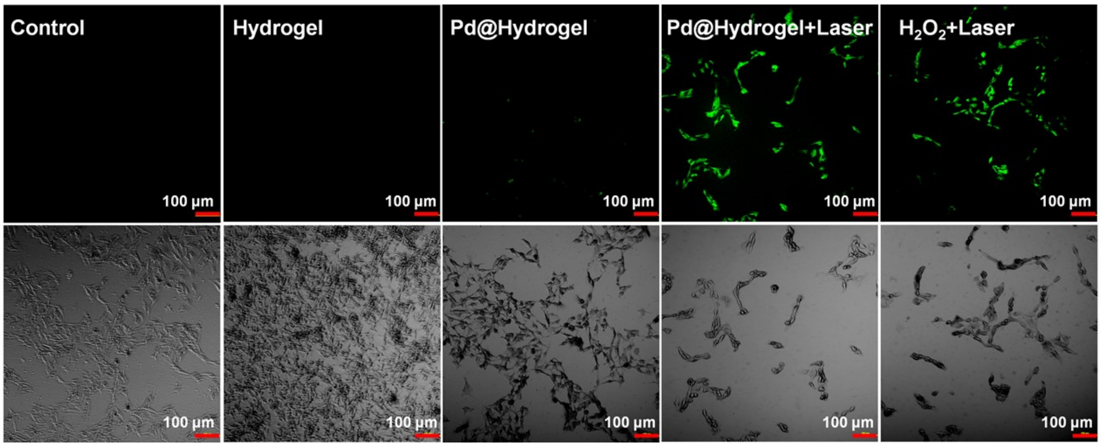

Fig S13. CLSM images of H_2DCFDA-stained $B_{16}F_{10}$ cells after different treatments, scale bar, 100 μm.

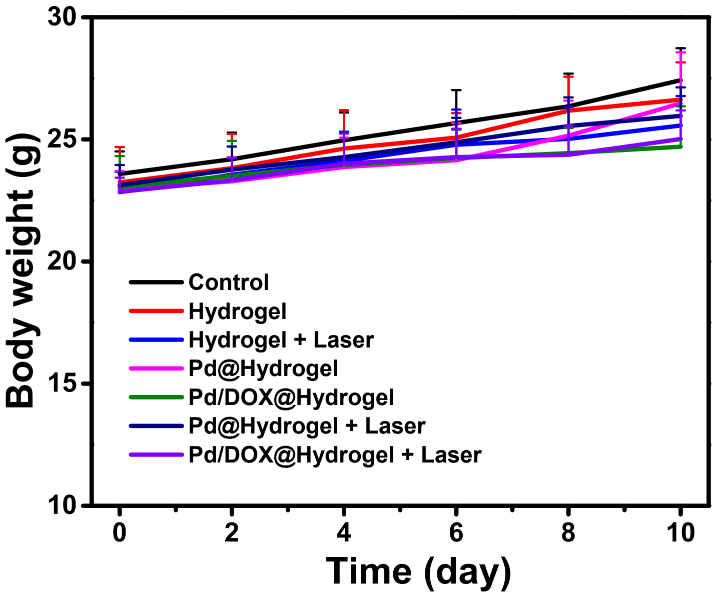

Fig S14. Body weight of balb/c mice from different treatments.

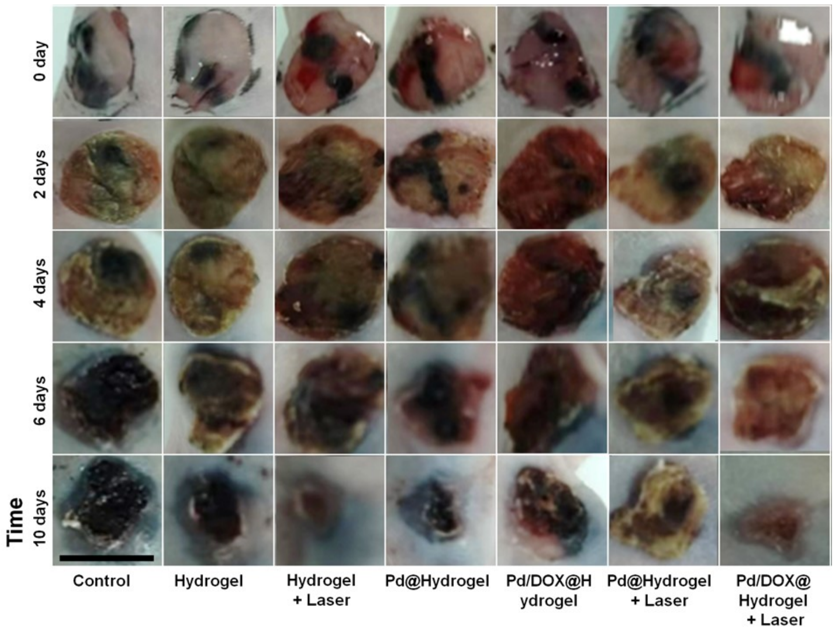

Fig S15. Photo of tumors and skin wounds at days 0, 2, 4, 6, and 10. Scale bar: 10 mm.

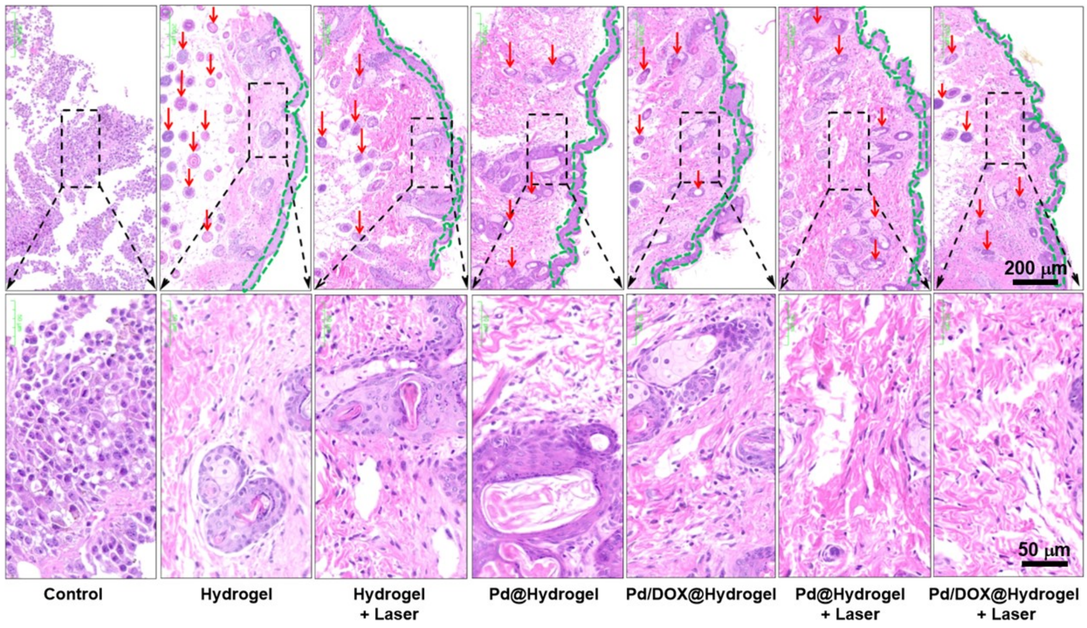

Fig S16. H&E-stained samples with new skin after different treatments. Scale bar: 200 μm (top) and 50 μm (beneath). Groups (control; hydrogel; Pd@Hydrogel; Hydrogel+Laser; Pd/DOX@Hydrogel; Pd@Hydrogel+Laser; Pd/DOX@Hydrogel+Laser).

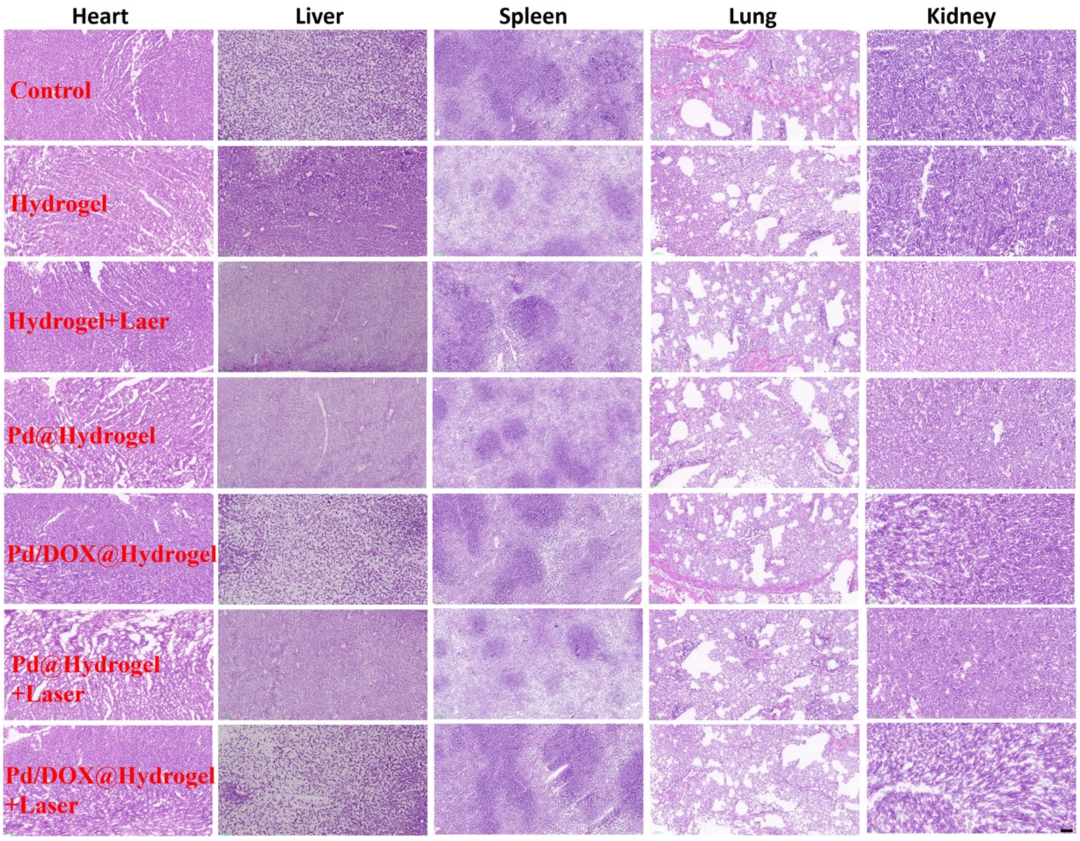

Fig. S17 H&E-stained major organs of mice 10 days after various treatments. Scale bar: 100 μm

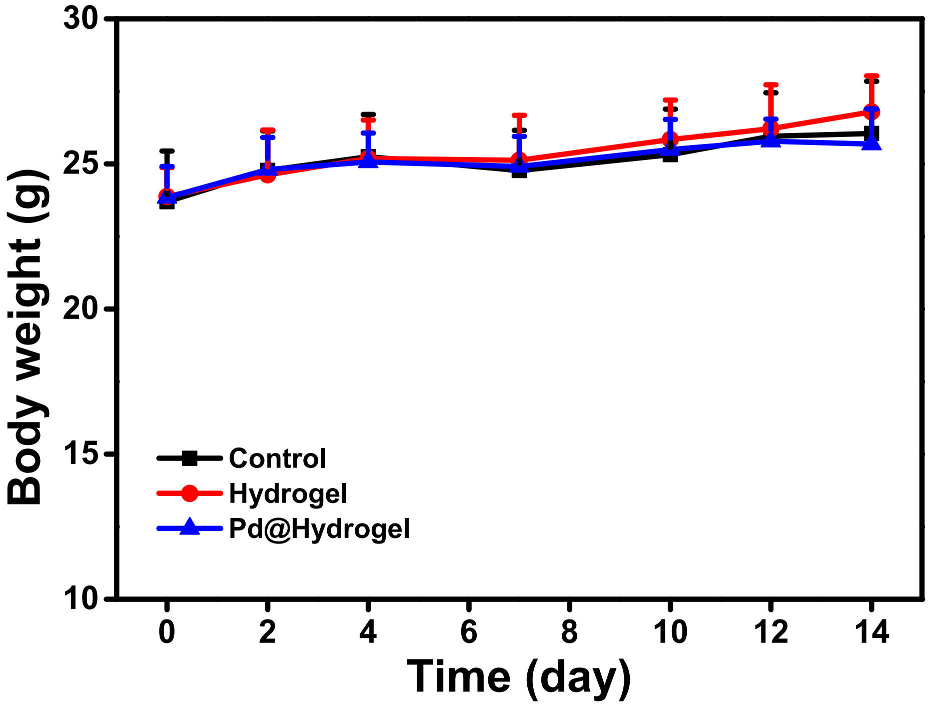

Fig S18. Body weight of mice (C57) from different treatments.
